# Association and haplotype analysis of candidate genes in five genomic regions linked to sow maternal infanticide in a white Duroc × Erhualian resource population

**DOI:** 10.1186/1471-2156-12-24

**Published:** 2011-02-09

**Authors:** Congying Chen, Zhuqing Yang, Yanying Li, Na Wei, Pinghua Li, Yuanmei Guo, Jun Ren, Nengshui Ding, Lusheng Huang

**Affiliations:** 1 Key Laboratory for Animal Biotechnology of Jiangxi Province and the Ministry of Agriculture of China, Jiangxi Agricultural University, Nanchang, 330045, PR China

## Abstract

**Background:**

Maternal infanticide is an extreme and failed maternal behavior, which is defined as an active attack on piglets using the jaws, resulting in serious or fatal bite wounds. It brings big economic loss to the pig industry and severe problems to piglets' welfare. But little is known about the genetic background of this behavior. Quantitative trait loci (QTL) for maternal infanticide were identified in a White Duroc × Erhualian intercross by a non-parametric linkage analysis (NPL) in our previous study. In this study, associations of 194 microsatellite markers used in NPL analysis with maternal infanticide behavior were further analyzed by transmission-disequilibrium test (TDT). On this basis, seven genes (*ESR*2, *EAAT*2, *BDNF*, *OXTR*, *5-HTR2C*, *DRD*1 and *GABRA*6) at five genomic regions were selected and further analyzed. Associations of single nucleotide polymorphisms (SNPs) and haplotypes in each gene with maternal infanticide behavior were evaluated.

**Results:**

Microsatellite markers on pig chromosome (SSC) 2, 13, 15, and X displayed significance at *P *< 0.05 by both TDT and NPL. Of the seven candidate genes, three *ESR*2 SNPs had nominal evidence for association (*P *< 0.05). Allele *A *at *EAAT*2 g. 233G > A and allele *T *at *DRD*1 g.1013C > G > T also showed evidence of overtransmission to infanticidal sows. In the overall tests of association of haplotypes, candidate genes of *ESR*2, *EAAT*2 and *DRD*1 achieved overall significance level (*P *< 0.05). Haplotype [A; A; G], [G; A; G], [A; G; G] and [C; C], respectively, from *ESR*2, *EAAT*2 and *DRD*1 showed higher frequencies to infanticidal sows (*P *< 0.05). Alleles among haplotypes and SNPs which showed an overtransmission to infanticidal sows were from White Duroc.

**Conclusions:**

From association tests of SNPs and haplotypes, *ESR*2, *EAAT*2 and *DRD*1 showed significant associations with maternal infanticide. This result supported the existence of QTL for maternal infanticide behavior on SSC1, SSC2 and SSC16.

## Background

Sows normally exhibit a series of maternal behaviors around parturition, but failure to establish normal maternal bonds with their newborn offspring occurs in some sows. An extreme behavior is maternal infanticide which is defined as an active attack of piglets that results in serious or fatal bite wounds around parturition or within 24 hours after parturition. From surveys of large commercial pig farms in Europe, the incidence of maternal infanticide was 8% [1] or 7-12% [2]. The incidence in primiparous sows is as high as 10-13% [2,3]. It not only brings a big economic loss to the pig industry, but also leads to poor animal welfare of piglets.

The aetiology of maternal infanticide in pigs is not clear. Environment, hormonal, parturition experience and genetic factors are all associated with maternal infanticide. The heritability of maternal infanticide differs between estimates of daughter/sire (0.12 - 0.25) and daughter/dam (0.5 - 0.9) [2]. It ranges between 0.4 and 0.9 and shows significant additive genetic effects in primiparous sows [1]. Quilter *et al. *(2007) detected four quantitative trait loci (QTL) for maternal infanticide on pig chromosomes (SSC) 2, 10 and X by non-parametric linkage analysis (NPL) in western commercial lines [4]. In our previous study, seven QTL were identified on SSC2, 6, 13, 14, 15 and X by NPL in a White Duroc × Erhualian F_2 _resource population [5].

Besides non-parametric linkage analysis, transmission-disequilibrium test (TDT) has also been widely used to investigate chromosome regions responsible for schizophrenia, bipolar disorder and other diseases in humans [6] and pigs [7,8]. So we herein used TDT to confirm the NPL results and discover other regions associated with maternal infanticide. The loci showing significance in both NPL and TDT analysis were considered to be the most interesting for further analyses.

Four functional related candidate genes including *excitatory amino acid transporter *2 (*EAAT*2), *brain derived neurotrophic factor *(*BDNF*), *oxytocin receptor *(*OXTR*) and 5-*hydroxytryptamine (serotonin) receptor 2C *(*5-HTR2C*) on the chromosome 2, 13 and X where QTL for maternal infanticide were identified by both NPL and TDT were chosen for further association analysis. A possible association between the A/G polymorphism at position 949 nt in *ESR*2 mRNA and susceptibility to maternal infanticide in western commercial sows was found by our European collaborators (personal communication, C. Sargent, University of Cambridge, UK). And based on important roles of estrogens in maternal behavior, the *ESR*2 gene was also analyzed for association with maternal infanticide behavior in the White Duroc × Erhualian resource population. A genomic region on SSC16 achieved near significance level (*P *= 0.067) in NPL analysis, so functional candidate genes of *Gamma-aminobutyric acid receptor A*6 (*GABRA*6) and *dopamine receptor D*1 (*DRD*1) were also investigated to confirm whether the QTL exited or not. Investigation of candidate genes in these regions could reveal causative genes for the maternal infanticide or linked markers, which would benefit marker-assisted selection (MAS) schemes for decreasing the incidence of maternal infanticide behavior.

## Results

### Significant loci identified by TDT

The significant loci revealed by TDT are presented in Table 1. Markers displaying significance at a corrected nominal value of *P *< 0.05 were discovered on SSC2, 9, 10, 11, 13, 15, 17 and X. Especially on the X chromosome, all 15 genotyped markers showed strong significant association with maternal infanticide (corrected *P *< 0.01). The significantly associated loci identified by both NPL and TDT analysis located on SSC2, 13, 15 and X.

**Table 1:** The genomic regions linked to pig maternal infanticide behavior by transmission-disequilibrium test (TDT)

chromosome	marker	position (cM)	Corrected *P *value	*P *value in NPL (Chen et al., 2009)
2	SW1879	103.70	0.02	4.90E-3
9	SW1349	144.10	0.02	0.10
10	SWC19	65.00	0.02	0.97
11	SW2413	106.30	0.01	0.40
13	S0283	83.50	0.03	0.04
15	SW1562	26.60	0.03	0.02
17	SWR1004	0.00	0.02	0.22
X	SW980	27.80	1.63E-10	1.00E-4
	SW1903	40.40	5.03E-10	1.78E-3
	SW2456	48.10	3.01E-10	9.29E-4
	UMNP1174	52.80	0.01	2.54E-3
	SWR1861	54.30	6.41E-3	3.26E-3
	UMNP71	55.60	3.85E-3	5.01E-3
	SW1994	55.80	6.63E-3	5.62E-3
	SW259	56.40	1.35E-10	5.57E-3
	SW1426	58.50	5.13E-3	9.10E-3
	SW1522	63.10	3.85E-3	0.01
	MCSI02	65.00	0.01	0.01
	SW1943	66.00	2.26E-10	0.01
	SW1608	71.70	6.41E-3	9.00E-4
	SW707	74.30	1.08E-10	7.87E-3
	SW2588	90.00	3.76E-10	0.02

### Significant markers of positional candidate genes

Comparative sequencing of founder animals of the resource population identified the polymorphisms of candidate genes. All exons, 5' promoter region and part of introns of *ESR*2, *BDNF*, *DRD*1, *OXTR *and *5-HTR2C *were screened. All sequences determined in this study have been deposited in GenBank with accession number HM754211, HM754212, HM754213, HM754214, HM754215, HM754216 and HM754217. The identified SNPs are given in Additional file 1, Table S1. Nine SNPs were detected in *ESR*2, including 6 in introns and 3 in exons. The mutation of A > G in exon 5 causes a change of methionine to valine. The other two coding SNPs are synonymous mutations. All 12 SNPs were found in introns of *BDNF*. For *DRD*1, a total of 3 SNPs were detected in exons, and all are synonymous mutations. Three SNPs were identified in exon 2 and five in introns of *OXTR*, and all exonic SNPs in this gene were synonymous mutations. Only 1 SNP was found in the *5-HTR2C *gene which located at intron 3. For *EAAT*2 and *GABRA*6 genes, only parts of sequences were screened for variants. Comparative sequencing revealed 10 polymorphisms in intron 7 and 2 synonymous polymorphisms in exon 9 of the *GABRA*6 gene. Twelve SNPs were identified in the intronic regions of the *EAAT*2 gene. Haplotypes were constructed for each candidate gene and their frequencies are shown in Table 2. Rare haplotypes (frequency < 0.05) were excluded from further analyses.

**Table 2:** Associations of the SNPs and haplotypes in *ESR*2, *EAAT*2, *BDNF*, *GABRA*6, *DRD*1, *OXTR *and *5-HTR2C *genes with sow maternal infanticide behavior

gene	Polymorphism	Allele or Haplotype^1^	Allele or Haplotype Frequency	Individuals of maternal infanticide				
				
				T	UT	DF	LRS	*P*
*ESR*2 (HM754215)	g.1170 G > A	*A*	0.341	40	24	1	4.043	0.044*
		*G*	0.659	24	40			
	g. 1202G > A	*A*	0.501	52	29	1	6.623	0.010*
		*G*	0.499	29	52			
	g. 2681G > T	*G*	0.534	50	28	1	6.290	0.012*
		*T*	0.466	28	50			
	-	*GGT*	0.457	10	30	2	5.993	0.049*
	-	*GAG*	0.143	7	2			
	-	*AAG*	0.361	23	8			
*EAAT*2 (HM754214)	g. 233G > A	*A*	0.693	43	20	1	8.594	0.003*
		*G*	0.307	20	43			
	g.489G > A	*A*	0.235	19	28	1	1.734	0.188
		*G*	0.765	28	19			
	g.1028G > A	*A*	0.238	20	29	1	1.662	0.197
		*G*	0.762	29	20			
	-	*AGG*	0.652	29	6	2	10.124	0.007*
	-	*GAA*	0.278	4	20			
	-	*GGG*	0.070	2	11			
*BDNF *(HM754212)	g.612C > A	*C*	0.748	27	19	1	1.391	0.238
		*A*	0.252	19	27			
	g.2723G > A	*G*	0.513	37	45	1	0.781	0.377
		*A*	0.487	45	37			
	g.3481A > G	*A*	0.594	33	31	1	0.063	0.803
		*G*	0.406	31	33			
	-	*AAA*	0.245	9	12	3	3.762	0.288
	-	*CAA*	0.241	19	8			
	-	*CGA*	0.108	4	9			
	-	*CGG*	0.394	16	19			
*OXTR *(HM754217)	g.509G > A	*G*	0.811	21	15	1	1.000	0.316
		*A*	0.189	15	21			
	g.1211T > C	*T*	0.487	26	35	1	1.333	0.248
		*C*	0.513	35	26			
	g.2271C > T	*C*	0.836	13	21	1	1.900	0.168
		*T*	0.164	21	13			
	-	*ACC*	0.193	15	8	3	2.350	0.503
	-	*GTT*	0.146	10	16			
	-	*GTC*	0.357	15	18			
	-	*GCC*	0.304	21	19			
*DRD*1 (HM754213)	g.1013C > G > T	*C*	0.500	47	35	2	9.485	0.009*
		*G*	0.424	28	46			
		*T*	0.076	7	1			
	g.1655C > T	*C*	0.795	26	17	1	1.898	0.168
		*T*	0.205	17	26			
	-	*GT*	0.205	5	17	3	10.068	0.018*
	-	*TC*	0.066	7	1			
	-	*CC*	0.511	30	18			
	-	*GC*	0.218	6	12			
*GABRA*6 (HM754216)	g. 176C > T	T	0.266	30	37	1	0.546	0.460
		C	0.734	37	30			
	g.444A > T	A	0.576	29	35	1	0.563	0.453
		T	0.424	35	29			
	g.1520G > A	T	0.590	29	34	1	0.397	0.529
		G	0.410	34	29			
	-	TTG	0.521	15	18	2	4.510	0.123
	-	CAT	0.257	15	17			
	-	CTG	0.132	11	3			
*5-HTR2C *(HM754211)	g.675C > T	A	0.606	15	7	1	2.909	0.088
		G	0.394	7	15			

Note: T: Transmission; UT: Untransmission; LRS: likelihood ratio statistic. The likelihood ratio statistic is minus twice the difference in log-likelihoods; *P*: P value from the overall test of association; DF: degree of freedom;

*: significant in statistics

1. Rare haplotypes were excluded from association study.

SNPs in which minor allele frequencies (MAF) were > 0.15 (Table 2) were chosen for genotyping animals in the resource population. Family-based association analysis was carried out on a total of 18 SNPs from seven candidate genes (Table 2). The significantly associated markers were found in *ESR*2, *EAAT*2 and *DRD*1. Allele *A *of *ESR*2 g.1170 G > A and g. 1202G > A (HM754215), allele *G *of *ESR*2 g. 2681G > T (HM754215) showed evidences of significant overtransmission of the major alleles to infanticidal sows (*P *< 0.05). Allele *A *of g. 233G > A in *EAAT*2 (HM754214) also showed a significant overtransmission of the major allele to infanticidal sows (*P *< 0.01). The overall *P *value of g.1013C > G > T in *DRD*1 (HM754213) also achieved < 0.01 significance level, and allele *C *more frequently transmitted to infanticidal sows. For *5-HTR2C*, although no allele showed significantly different transmission between infanticidal sows and normal sows, allele A had a higher frequency transmitted to infanticidal sows (*P *= 0.088). Interestingly, through checking the origin of alleles, we found that all alleles which had overtransmission to infanticidal sows were from White Duroc.

To further support the evidences of association, the overall tests of association were also performed on haplotypes. Only *ESR*2, *EAAT*2 and *DRD*1 showed significantly overall *P *value (Table 2; *P *< 0.05). Haplotypes [A;A;G] and [G;A;G] from *ESR*2, [A;G;G] from *EAAT*2, and [C;C] from *DRD*1 showed an overtransmission to infanticidal sows. All these haplotypes were from the breed of White Duroc.

## Discussion

Maternal infanticide behavior, a lacking of a clear Mendelian inheritance pattern, could be caused by several genes of low to moderate penetrance, TDT or NPL analysis are hence a more robust and successful alternative to identify susceptible loci for the abnormal behavior. As in other studies [7,9], we found distinct linkage results between TDT and NPL [5]. NPL test for markers linked to genes affecting disease status on the basis of identity-by-descent, and is more powerful when the marker density is low. TDT is a fine mapping method requiring linkage disequilibrium (LD) between causative mutations and the linked markers [8,10]. It shows greater power when markers are very close to the causative gene. In this study, the discrepancy between TDT and NPL may be caused by the low marker density and the multiple marker alleles.

Seven candidate genes, of which *EAAT*2, *BDNF*, *OXTR *and *5HTR2C *are positional and functional candidate genes, and *ESR*2, *DRD*1 and *GABRA*6 are functional candidate genes, were chosen from five genomic regions including SSC1, SSC2, SSC13, SSC16 and SSCX for further analyzing to find candidate markers linked to sow maternal infanticide behavior. Significant associations were identified on all three *ESR*2 SNPs, and the overall test of association of the haplotypes also achieved the significance level. Haplotypes of [G;G;T] and [A;A;G] showed very significant transmission disequilibrium between normal and infanticidal sows (*P *< 0.01). Estrogen and progesterone are important in the initiation of maternal behavior [11]. Estrogens have generally excitatory effects [12] and considerable regulatory influence on systems mediating anxiety and mood [13]. Sows with high ratio of estrogen to progesterone in late pregnancy showed increased savaging to their piglets [14]. Estrogen's actions could be mediated by estrogen receptor 1 or 2 (α or β). *ESR*1 is critical for reproduction function, and *ESR*2 is critically involved in regulating reproductive behaviors, brain development and procession of emotional behavior (anxiety-related behavior e.g.) [15]. This result was also consistent with the results in western commercial sows (personal communication, C. Sargent, University of Cambridge, UK), in which an A/G polymorphism at position 949 nt of *ESR*2 mRNA was associated with maternal infanticide. However, no QTL was detected on SSC1 in whole genome QTL mapping. It could be caused by low marker density tested on this chromosome.

Another significant association was found on *EAAT*2 gene. Extreme significance level was achieved both in analyses of g. 233G > A and haplotypes (*P *< 0.01, Table 2). Although the significant microsatellite marker and *EAAT*2 gene were not in the same genomic region on SSC2, this result should confirm the existence of QTL on this chromosome owning to large linkage disequilibrium in F_2 _population. *EAAT*2 is responsible for the majority of glutamate uptake in the brain and its disregulation has been associated with multiple psychiatric and neurological disorders [16]. Its expression in infanticidal sows from the current population is down-regulated (Data from cDNA microarray analysis, unpublished). Combined with the association result in this study, it is a strong candidate gene for maternal infanticide.

The last significant association was identified on *DRD*1 gene. *DRD*1 encodes the D1 subtype of the dopamine receptor. D1 receptors regulate neuronal growth and development, mediates some behavioral responses and schizophrenia [17,18]. Given the differently transmitted frequency of the *DRD*1 alleles and its important roles in behavioral responses, *DRD*1 could be an important candidate gene influencing sow maternal infanticide behavior. The significant association of *DRD*1 with maternal infanticide indicated that a causative gene may exist within this chromosome region. Only five microsatellite markers on SSC16 were used in the whole genome QTL scanning, therefore the absence of a QTL on this chromosome may be due to low marker density.

Interestingly, the alleles both in SNPs and haplotypes which had overtransmission to infanticidal sows were from White Duroc. This result was also consistent with the previous reports about maternal ability of Chinese and Western pig breeds. The Chinese Erhualian pig breed is the most famous Chinese indigenous pig breed for its excellent reproductive performance. Van der Steen and de Groot (1992) found that Meishan piglets have 5% advantage in survival rate than large white piglets due to good maternal behavior [19]. The Chinese Meishan breed exhibits good maternal characteristics with resulting even heavier piglets at weaning and lower incidence of aggressive behavior towards their offspring than European white sows [20].

The other candidate genes, such as *BDNF*, *OXTR *and *GABRA*6, which play important roles in the phenotypes similarly to maternal infanticide behavior, for example anxiety-related behavior [21], mood disorders [22], depression-related traits [23] and pressure response [24], did not show any significant association with maternal infanticide behavior in this study. So these genes are not the candidate genes for maternal infanticide in the White Duroc × Erhualian resource population, or more SNPs in these genes needed to be evaluated. Especially for *OXTR *gene, in rats, females having a significantly high expression level of oxytocin receptors in several areas of brain showed more maternal care to offspring [25], and the oxytocin receptor gene located proximal to a QTL on SSC13 detected by both NPL and TDT. However, the QTL on this chromosome only achieved *P *< 0.05 significance level, false discovery can not be excluded.

For chromosome X, similarly to the results in NPL [9] and western commercial pig lines [8], all 15 microsatellite markers showed very significant association with maternal infanticide behavior in the TDT analysis. 5-*HTR2C *shows close relationship with anxiety-like behavior and major mental disorders including schizophrenia, bipolar disorder and major depression [26,27]. In this study, allele *A *of g.675C > T (HM754211) was preferably transmitted to infanticidal sows compared with allele C. The *P *value did not achieve statistical significant level due to the small population size, or more SNPs in this gene or more candidate genes need to be analyzed.

## Conclusions

Microsatellite markers on SSC2, 13, 15 and X were identified to associate with maternal infanticide both by NPL and TDT analysis. From the overall association test of SNPs and haplotypes in seven candidate genes, *ESR*2, *EAAT*2 and *DRD*1 gene showed significant association with sow maternal infanticide. Given the extensive linkage disequilibrium in a F_2 _population, although some candidate genes showed highly significant association with maternal infanticide behavior, they are more likely linked genes rather than causal genes. The association results provided the evidence for locating the QTL, and would provide some useful information for future studies directing at the identification of the causative mutations.

## Methods

### Animals

A F_2 _resource population was developed using two divergent pig breeds of White Duroc and Chinese Erhualian. Two White Duroc boars and 17 Erhualian sows were crossed as founder animals to produce F_1 _animals, and 59 F_1 _sows were randomly mated with 9 F_1 _boars to produce 1912 F_2 _individuals, of which 288 F_2 _sows from 83 families were well phenotyped for maternal behaviors around parturition over three continuous farrowings. Each sow was housed in a pen of 2 × 2.5 meters with a concrete floor, solid walls of 1 meter high during the giving birth. The feeding and management of the sows were described in detail in Chen et al. 2008 [3]. All animal procedures were conducted according to the guidelines for the care and use of experimental animals established by the Ministry of Agriculture of China.

### Phenotype recording

Sow maternal behavior was observed and recorded from 6 hours before parturition to 24 hours after giving birth. The methods for phenotypic records were described previously [3]. In brief, from the first piglet's birth, sow maternal infanticide was recorded, which was defined as an apparently deliberate attack on one or more piglets that resulted in a serious wound or the death by biting of at least one piglet. Sows with infanticide behavior were marked 1, conversely marked 0. The phenotypic records were performed on 288 F_2 _sows at their first to third farrowings. A total of 47 sows showed maternal infanticide behavior.

### Identification of polymorphisms in candidate genes

Genomic DNA was isolated from porcine ear tissues using proteinese K digestion followed by phenol/chloroform extraction and precipitation with ethanol. DNA of founder animals or nine F_1 _boars was used for detecting polymorphisms. Primers were designed based upon the genomic DNA sequence at Ensemble (Sscrofa9, Apr 2009) [28] using software Primer 5.0. Primer sequences, amplified gene region, length of amplicons and their annealing temperature are shown on Additional file 1, Table S1. Amplification was performed in a 20 μl reaction volume containing 40 ng genomic DNA template, 0.4 μl of each dNTP (0.2 mM), 0.3 μl of each primer (0.2 μM) and 2 U of Taq DNA polymerase (Takara, Dalian, China) at 94°C for 5 min, 35 × (94°C for 30 sec, annealing temperature for 30 sec, 72°C for 45 sec), 72°C for 7 min on PTC-200 Thermal Cyclers (Bio-RAD, Waltham, USA). After purified with the QIAquick DNA Purification Kit (Qiagen, Hilden, Germany), PCR products were bidirectly sequenced by BigDye Terminator v3.1 on an ABI3130XL genetic analyzer (Applied Biosystems, Foster, USA). The obtained genomic sequences were analyzed for SNPs using Chromas or SeqMan.

### Genotyping

The selection and genotyping of 194 microsatellite markers in the whole scan were described in detail in Chen et al. 2009 and Guo et al. 2009 [9,29]. Except *5-HTR2C *and *DRD*1 in which only one and two identified SNPs were genotyped, respectively, three SNPs from each candidate gene were selected according to their polymorphic information (MAF > 0.15) and genotyped in the resource population. SNPs of *EAAT*2 and *GABRA*6 were genotyped by PCR-RFLP using primers in Additional file 1, Table S2. For the SNP of *GABRA*6 g.176C > T and the SNP of *EAAT*2 g.233G > A, a primer mutagenesis assay was established for these sites. For the SNP of *GABRA*6 g.176C > T, a mismatch nucleotide (G) was incorporated at position 29 of the forward primer to amplify a fragment of 250 bp, and for the SNP of *EAAT*2 g.233G > A, the mismatch nucleotide (G) was located at position 28 of the forward primer (Additional file 1, Table S2). Five microliter PCR products were digested in a volume of 15 μl containing 2 U restriction enzymes (NEB, Beijing, China) and 1 × supplied buffer by incubating at 37°C water for 6h. The restriction fragments were separated on 2% agarose gels in 1×TAE buffer at constant voltage of 180 V. The gels were stained with ethidium bromide and visualized on an UV transilluminator.

The *ESR*2, *BDNF*, *DRD*1, *OXTR *and *5-HTR2C *SNPs were genotyped using the SNaPshot kit (Applied Biosystems, Foster, USA) according to the manufacturers' protocol. Briefly, PCR reaction was performed as described above. Three microlitre PCR products were purified with 1 μl enzyme of ExoSap-IT (USB, USA) at 37°C for 15 min, followed by 80°C for 15 min. Three SNPs in each gene were genotyped in a single SNaPshot reaction. The SNaPshot reaction was performed at 5 μl mixture containing 2 μl SNaPshot Multiplex Ready Reaction Mix, 0.5 μl mixed SNaPshot primers and 2.5 μl mixed purified PCR products at 35 × (96°C for 10 sec, 50°C for 10 sec, 60°C for 30 sec) on PTC-200 Thermal Cyclers (Bio-RAD, Waltham, USA). Primers for PCR and SNaPshot extension assays are listed in Additional file 1, Table S3. The SNaPshot reaction product was purified with 1 U CIP (NEB, Beijing, China) at 37°C for 1 h, followed by 75°C for 15 min. Eight microlitre Hi-Di were mixed with 0.5 μl purified SNaPshot product, 0.4 μl GeneScan120 LIZ size standard and 1.1 μl distilled water. After denaturalized at 95°C for 5 min, the mixture was placed immediately on ice, and then loaded on an ABI 3130XL genetic analyzer for data collection. The SNP genotypes were recorded by GeneScan version 4.0.

### Statistical analysis

The classical transmission-disequilibrium test (TDT) and the sib transmission-disequilibrium test (S-TDT) were performed with TDT-STDT program version 1.1 under defaulted settings [30,31] for the 194 microsatellite markers. Bonferroni correction was used to remove the effect of multiple tests and determine the significant *P *value. SNP genotypes were corrected for Mendelian errors using FBAT software. To eliminate stratification effects, family-based association analysis of each SNP marker and haplotype was carried out using transmission-disequilibrium test (TDT) via the TDTPHASE software in UNPHASED package [32]. In this analysis, the overall test of association was the likelihood ratio test. The log-likelihoods for the null and alternative hypotheses were displayed. The p-value was the probability of observing a likelihood ratio statistic at least as large as this one, if the null hypothesis were true [32]. Only complete trios and one trio per multiplex family were included. Linkage disequilibrium values were measured from genotype data of F_2 _individuals using 2LD software [33]. Individuals' haplotypes were constructed using Simwalk2 software [34]. Rare haplotypes at frequencies lower than 0.05 were excluded from the test.

## Authors' contributions

Conceived and designed the experiments: LSH. Performed the experiments: CYC, ZQY, YYL, NW and PHL. Analyzed the data: NSD, CYC, ZQY, YYL and YMG. Wrote and revised the paper: CYC, LSH. Provided comments and suggestions for the manuscript: JR. All authors read and approved the final manuscript.

## Supplementary Material

Additional file 1 sequences and assay conditions of primers for SNPs identification and genotyping Click here for file
